# Nirsevimab Administration and RSV Hospitalization in the 2024-2025 Season

**DOI:** 10.1001/jamanetworkopen.2025.33535

**Published:** 2025-09-24

**Authors:** Jonathan H. Pelletier, Sarah Z. Rush, Eric Robinette, Danielle E. Maholtz, Michael T. Bigham, Michael L. Forbes, Steven L. Shein, Todd J. Karsies, Christopher M. Horvat

**Affiliations:** 1 Division of Critical Care Medicine, Department of Pediatrics, Akron Children’s, Akron, Ohio; 2 Department of Pediatrics, College of Medicine, Northeast Ohio Medical University, Rootstown; 3 Division of Hematology and Oncology, Department of Pediatrics, Akron Children’s, Akron, Ohio; 4 Division of Infectious Disease, Department of Pediatrics, Akron Children’s, Akron, Ohio; 5 The Rebecca D Considine Research Institute, Akron Children’s, Akron, Ohio; 6 Division of Critical Care Medicine, Department of Pediatrics, Rainbow Babies & Children’s Hospital, Cleveland, Ohio; 7 Division of Critical Care Medicine, Department of Pediatrics, Nationwide Children’s, Columbus, Ohio; 8 Division of Pediatric Critical Care Medicine, Department of Critical Care Medicine, UPMC Children’s Hospital of Pittsburgh, Pittsburgh, Pennsylvania

## Abstract

This cohort study examines rates of hospitalization associated with respiratory syncytial virus (RSV) among infants by nirsevimab administration during the 2024-2025 season.

## Introduction

Respiratory syncytial virus (RSV) is a leading cause of hospitalization for respiratory infections in infants. Hospitalization risk factors include younger age, prematurity, medical complexity, and lower socioeconomic status.^1^ Since 2023, the US Centers for Disease Control and Prevention has recommended nirsevimab to prevent RSV for all infants younger than 8 months entering their first RSV season and high-risk infants aged 8 to 19 months between October and March, excluding those born to vaccinated mothers.^2^ Available nirsevimab data are from the 2023 to 2024 RSV season with limited drug supply.^3^ We conducted a cohort study of the 2024 to 2025 RSV season, hypothesizing that nirsevimab would be associated with reduced RSV-associated hospitalization.

## Methods

This cohort study was deemed exempt from review and informed consent by the Akorn Children’s institutional review board because the study was secondary use of an anonymized data source. We followed the Strengthening the Reporting of Observational Studies in Epidemiology (STROBE) reporting guideline for observational studies.

We conducted this retrospective cohort study using the Epic Systems Cosmos dataset,^4^ created in collaboration with a community of health care systems using Epic Systems software, representing more than 300 million patients from more than 1700 hospitals and 40 000 clinics from all 50 states, the District of Columbia, Canada, Lebanon, and Saudi Arabia. We included infants born between February 1, 2024, and January 31, 2025, to ensure at least 2 months within the 2024 to 2025 RSV season, defined as October 1, 2024, to March 31, 2025. We did not include infants aged 8 to 19 months due to data granularity. The exposure was nirsevimab treatment, and the outcome of interest was RSV-associated hospitalization.^5^ We excluded infants with missing maternal information (linked on birth events), infants born to mothers who received an RSV vaccine 14 to 280 days prior to delivery, infants who died without hospitalization, infants who received nirsevimab or had RSV-associated hospitalization during the 2023 to 2024 RSV season (before April 1, 2024), and infants who did not have well-child visits after September 30, 2025, to reduce loss to follow-up. We stratified infants according to nirsevimab administration. Continuous variables were compared with 2-sided Wilcoxon rank sum test, and categorical variables were compared with 2-sided Pearson χ^2^ tests. *P* < .05 was considered statistically significant. We analyzed the daily RSV-associated hospitalization rate using generalized additive models. We trained multistate Cox proportional hazards models with and without adjustment for infants’ chronologic age, gestational age, Social Vulnerability Index, and cardiovascular, neurologic, and respiratory complex chronic conditions.^6^ We transitioned patients from untreated to treated on the day of nirsevimab administration and reported hazard ratios (HRs). Data were analyzed using R software version 4.5.0 (R Project for Statistical Computing).

## Results

We identified 798 470 infants born between February 1, 2024, to January 31, 2025. We excluded 388 747 infants (256 494 with missing maternal information; 75 107 with maternal vaccination; 472 who died; 11 946 who received nirsevimab before April 1, 2024; 73 with RSV-associated hospitalization before April 1, 2024; and 44 655 with no well visits after September 30, 2025). A total of 409 723 infants (median [IQR] age, 8 (5-10) months; 209 543 [51.1%] male) were included in the study, of whom 194 422 (47.5%) received nirsevimab. Demographics are shown in the Table. Daily nirsevimab administrations and RSV-associated hospitalizations are shown in the Figure. Overall, 850 of 194 422 treated patients (0.4%) vs 2535 or 215 301 untreated patients (1.2%) underwent RSV-associated hospitalization (*P* < .001). The maximum estimated daily hospitalization rate was 2.90 (95% CI, 2.42-3.38) per 100 000 treated infants vs 13.84 (95% CI, 13.16-14.53) per 100 000 untreated infants. Results of Cox proportional hazard modeling are shown in the Figure. The unadjusted and adjusted hazard ratios for RSV-associated hospitalization after treatment with nirsevimab were 0.29 (95% CI, 0.26-0.32) and 0.23 (95% CI, 0.21-0.26), respectively.

**Table.** Study Demographics

Characteristic	Participants, No. (%)			*P* value
	Overall (N = 409 723)	Receipt of nirsevimab		
		No (n = 215 301)	Yes (n = 194 422)	
Age, median (IQR), mo	8 (5-10)	9 (6-11)	7 (5-9)	<.001^a^
Sex				
Male	209 543 (51.1)	110 106 (51.1)	99 437 (51.1)	.98^b^
Female	200 180 (48.9)	105 195 (48.9)	94 985 (48.9)	
CCC				
Cardiovascular	10 773 (2.6)	4540 (2.1)	6233 (3.2)	<.001^b^
Respiratory	477 (0.1)	143 (0.1)	334 (0.2)	<.001^b^
Neurologic	2132 (0.5)	909 (0.4)	1223 (0.6)	<.001^b^
Gestational age				
Median (IQR), wk	39 (38-40)	39 (38-40)	39 (38-40)	<.001^a^
Unknown, No.	611	352	259	NA
Social Vulnerability Index				
Median (IQR)	67 (38-87)	68 (40-87)	66 (37-87)	<.001^a^
Unknown	4718	2775	1943	NA
RSV-associated admission	3385 (0.8)	2535 (1.2)	850 (0.4)	<.001^b^
RSV-associated ICU admission	1089 (0.3)	765 (0.4)	324 (0.2)	<.001^b^
RSV-associated admission requiring intubation	139 (<0.1)	90 (<0.1)	49 (<0.1)	.004^b^

Abbreviations: CCC, complex chronic condition; ICU, intensive care unit; NA, not applicable; RSV, respiratory syncytial virus.

^a^ Wilcoxon rank sum test.

^b^ Pearson χ^2^ test.

**Figure.**
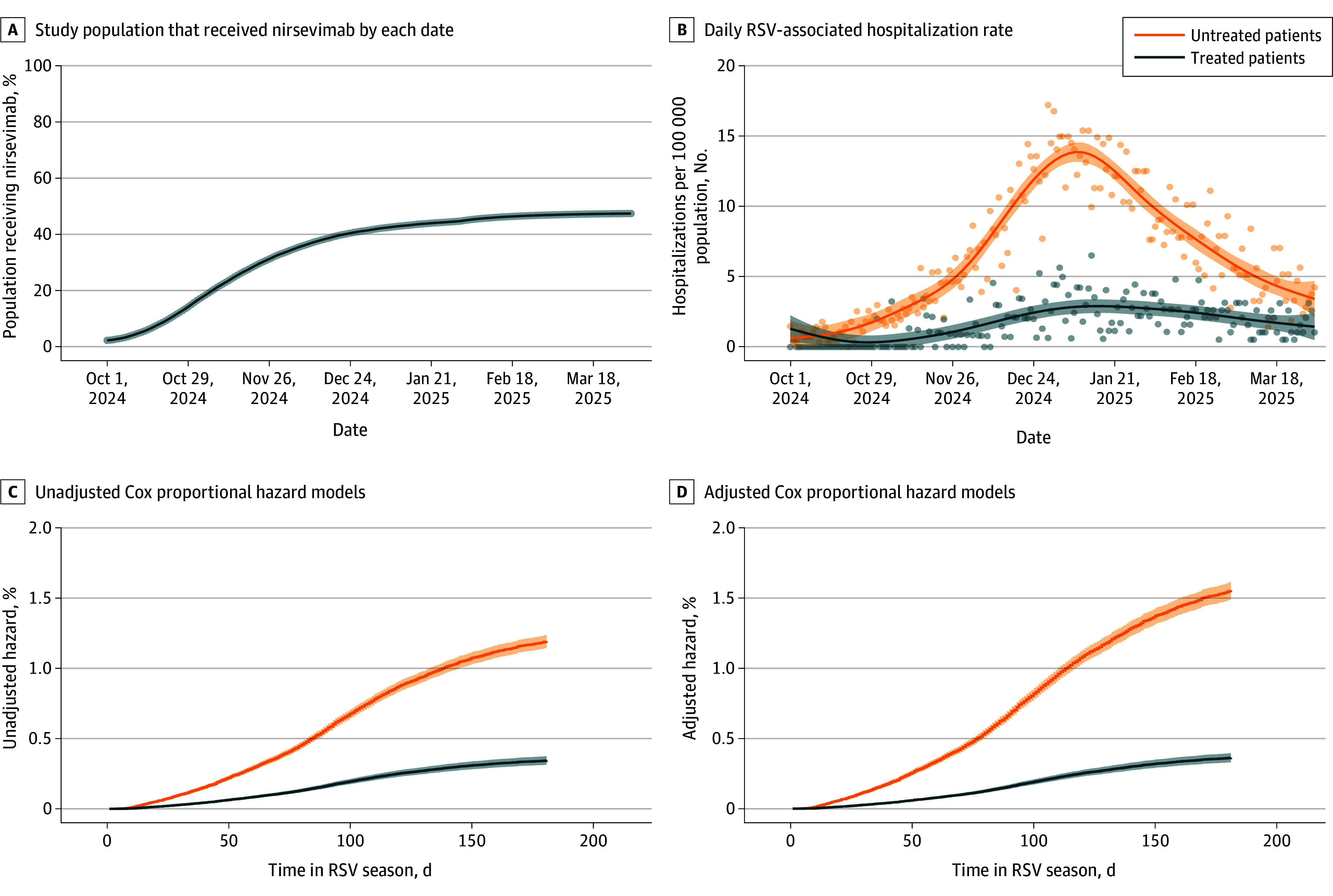
Daily Nirsevimab Administration and RSV-Associated Hospitalization In panel B, dots indicate actual daily hospitalization rate, and lines indicate estimation from a generalized additive model. In C and D, lines indicate model estimate. Shaded regions in all panels denote 95% CIs. RSV indicates respiratory syncytial virus.

## Discussion

To our knowledge, this cohort study is the first study of nirsevimab during the 2024 to 2025 RSV season and has a larger sample than a 2025 meta-analysis.^3^ The reduction in RSV-associated hospitalization reported in this study is similar to the 2023 to 2024 RSV season.^3^ Although we only included infants who received well-child care within the health system during the 2024 to 2025 RSV season, this study is still limited by the possibility of nirsevimab administration at facilities not included in this dataset or loss to follow-up. It is further limited by the possibility of missing maternal RSV vaccine information. All of these may bias toward the null. Despite these limitations, this study and prior analyses offer strong evidence that nirsevimab remains an effective tool to reduce RSV-associated hospitalization. Public health groups should continue to recommend nirsevimab.

## Notes

Supplement.
Data Sharing Statement
