# Supplementary material for: Nirsevimab Administration and RSV Hospitalization in the 2024-2025 Season
Source: JAMA Netw Open. 2025 Sep 24;8(9):e2533535. doi: 10.1001/jamanetworkopen.2025.33535 (PMC12461420; doi:10.1001/jamanetworkopen.2025.33535)
Supplement: Supplement. — Data Sharing Statement [file jamanetwopen-e2533535-s001.pdf]

Data Sharing Statement

Pelletier. Nirsevimab Administration and RSV Hospitalization in the 2024-2025 Season. *JAMA Netw Open*. Published September 25, 2025. doi:10.1001/jamanetworkopen.2025.33535

Data
Data available: No

Additional Information
Explanation for why data not available: The data used in this study is part of Epic Cosmos. Interested centers can visit https://cosmos.epic.com for information regarding joining.
